# First Report of Gynandromorphism in *Rhipicephalus microplus* (Acari: Ixodidae) in El Salvador

**DOI:** 10.1007/s11686-026-01327-9

**Published:** 2026-06-25

**Authors:** Marvin Stanley Rodriguez Aquino, Esther Noemí Juárez Alvarado, Francisco Javier Castaneda Lúe, Pamela Michelle Cornejo Rivas, Melissa S. Nolan, Lídia Gual-Gonzalez

**Affiliations:** 1 Centro de Investigación y Desarrollo en Salud (CENSALUD), Universidad de El Salvador, San Salvador, El Salvador; 2 Department of Epidemiology and Biostatistics, Arnold School of Public Health, University of South Carolina, Columbia, USA; 3 Institute for Infectious Disease Translational Research, University of South Carolina, Columbia, USA; 4 Department of Public Health Sciences, College of Behavioral, Social, and Health Sciences, Clemson University, Clemson, USA

**Keywords:** Gynandromorphism, Ticks, Rhipicephalus microplus

## Abstract

**Purpose:**

Gynandromorphism is a developmental anomaly characterized by the simultaneous presence of male and female sexual morphological traits within the same individual. This study documents a case of gynandromorphism in the cattle tick *Rhipicephalus microplus*(Canestrini 1888) in El Salvador.

**Methods:**

The specimen was collected from a bovine belonging to the Experimental and Practical Station of the Faculty of Agronomic Sciences of the University of El Salvador, located in the municipality of San Luis Talpa, Department of La Paz.

**Results:**

The reported gynandromorph of *R. microplus* exhibits bilateral gynandromorphism, displaying primarily female characteristics on the left side and male characteristics on the right.

**Conclusion:**

This is the first report of a tick with gynandromorphism in El Salvador. Further studies are warranted to understand the implications of such anomalies and their veterinary and public health relevance.

## Introduction

 Gynandromorphism, sometimes presented as sexual chimerism, is a biological condition that can originate through diverse mechanisms, such as hormonal alterations [5], genetic anomalies during chromosomic segregation [14] or fertilization of binuclear oocytes as described by [3]. When the male and female sexual characteristics distribution occurs in a comparable way in both body halves, the organism is classified as bilateral gynandromorph. If the alteration emerges during more advanced stages in the embryonic development or cellular division, the symmetry may be irregular or only partial, reaching more complex patterns expressed with axial configurations or mosaicism, which can evolve from different etiologic processes [3].

Gynandromorphism is not an uncommon developmental abnormality in ticks. This phenomenon has been previously documented in some free-living genera of ticks, particularly *Amblyomma sp.* and *Hyalomma sp.* [11]. In terms of gynandromorphism reports among the genus *Rhipicephalus* in Neotropical regions, there are some documented cases among brown dog tick, *Rhipicephalus sanguineus* sensu lato [7, 10, 13]. Similarly, there have been instances reported in *R. (Boophilus) microplus*, corresponding to a category classified as semi-divided gynandromorphism called “ginandromorfismo semi-partido”, where both male and female characteristics are expressed as irregular features, mostly as presenting male parts on a female body [6]. This short communication describes the first reported instance of gynandromorphism in ticks in El Salvador in *R. microplus*.

## Methods

In May 2021, our team members from the University of El Salvador’s Health Discovery and Investigation Center, (Centro de Investigación y Desarrollo en Salud: CENSALUD), began a tick surveillance initiative aimed at creating a biorepository of ticks from across the different departments in El Salvador. The goal is to obtain a large collection that will be used to detect veterinary and medically important vectors and associated pathogens across the country. Since 2021, we have visited facilities across seven departments on a convenience basis, at least every 6 months. The sampling was still ongoing during the development of this report.

In October 2025, we visited the university’s agricultural research facility (Estación Experimental y de Prácticas, Facultad de Ciencias Agronómicas) located in the municipality of San Luis Talpa, Departamento de La Paz (13°28’22.4"N, 89°05’46.1"W). During the sampling we examined 23 animals (12 cows, 10 goats and one dog) and a human, as well as performed environmental sampling of questing ticks around the perimeter of the facility. While examining the animals, an unusual specimen was found attached to the udder of a Holstein Brown Swiss cattle (*Bos primigenius taurus*). The tick was removed using fine forceps and preserved in a vial containing 70% ethanol and 5% glycerol. We performed taxonomic identification following the “Garrapatas Ixodidae de Panamá" identification key [2] using a stereomicroscope Leica EZ4E (Leica Microsystems Inc, Wetzlar, Germany). The images were taken using a smartphone placing it in the ocular lens of the microscope.

## Results

**Fig. 1:**
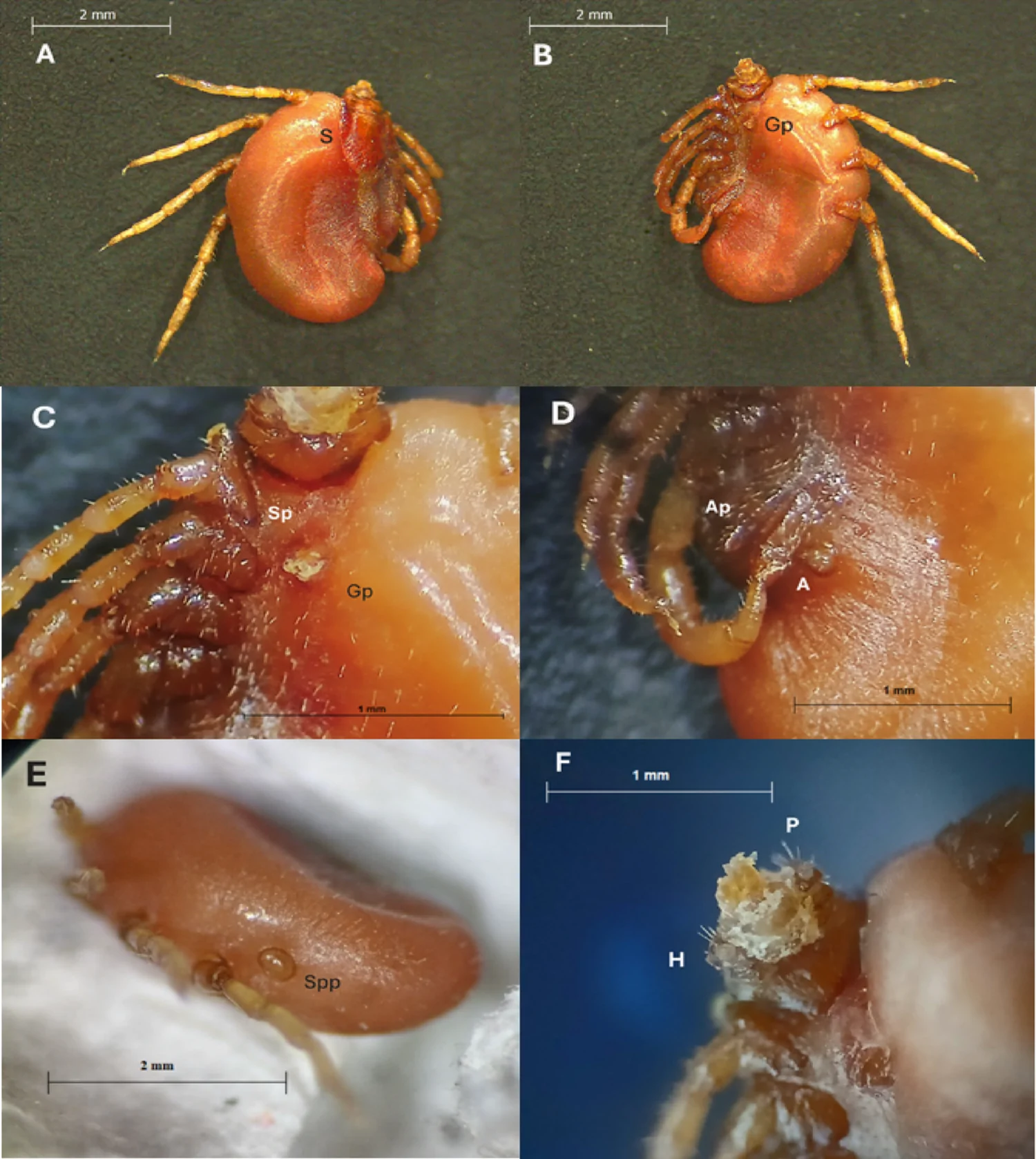
Morphologic characteristics of gynandromorphic R. microplus.** A** Dorsal view,** B**–**D** Ventral views.** E** Lateral view.** F** Capitulum. S=scutum, Gp=genital pore, Sp=spur, Ap=adanal plate, A=anal pore, Spp=spiracular plate, H=hypostome, P=palps

In October 2025, we collected 192 ticks from the university’s agricultural research facility in San Luis Talpa, La Paz. A single morphologically unique tick was collected and taxonomically identified as *R. microplus*. The tick presented evidence of gynandromorphism with an asymmetric division. As observed in Fig. 1A, the tick displayed a longitudinal division from the base of the capitulum to the posterior end. On the left side dorsal view, the tick exhibited predominantly female characteristics, including a scutum, and male characteristics were observed on the right side.

The specimen presented an anal groove slightly over the female side, and a genital pore located medially. It is worth noting that these medial features leaned to the male side due to the engorgement of the female side (Fig. 1B). On the male side, adanal plates often seen in male ticks were observed; whereas no caudal pedunculus was identified (Fig. 1D). Moreover, the coxae present on the male side were short and displayed pointed spurs on the first coxa. Comparatively, on the female side, the spurs were short and rounded (Fig. 1C). The tick presented spiracular plates on both sides (Fig. 1E). The capitulum did not show division differences, unlike the idiosoma (Fig. 1F), however the small, hexagonal, basis capituli presented female characters with palps shorter than the hypostome.

## Discussion

There are limited reports of gynandromorphism in *R. microplus* (previously, *Boophilus microplus*) compared to other tick species, and to this date, it has never been reported in ticks from El Salvador [6, 8]. Loomis and Stone [8], reported a *R. microplus* with gynandromorphism collected from a Shorthorn calf in Australia. This tick had female attributes on the left side and male characteristics on the right side, without a complete bilateral division into female and male halves, the tick showed predominantly male characteristics on the posterior end [8]. Comparatively, our findings showed asymmetric distribution with predominantly female characteristics. Another instance of gynandromorphism in *R. microplus* was described in a collection from a Shri Bareilly Gowshala calf in India [6]. This tick possessed a scutum in its normal location and a partial conscutum in a posterior lateral position to the left of the idiosoma. Although the tick presented male characteristics, the genital pore showed only female characteristics, different from our report where we observed a division between both sexes splitting the genital pore.

While morphological abnormalities are often reported in ticks, gynandromorphism remains rare. Domínguez and Bermúdez [4], recently reviewed a large tick collection of 6,624 hard ticks from the Instituto Conmemorativo Gorgas de Estudios de la Salud in Panamá. Among the 6,624 specimens, they identified 255 ticks with morphological anomalies, and only two *Amblyomma dissimile* specimens showed gynandromorphism. In the review, authors suggested that gynandromorphism and other general abnormalities are less frequent than local malformations, contrary to a report from Brazil where authors stated that gynandromorphism was the predominant anomaly [4, 9]. Nonetheless, reports of these type abnormalities seem to be rarer in *Rhipicephalus* ticks than other genus like *Amblyomma*, and the general underreporting of morphological anomalies in ticks could bias these interpretations highlighting the need of further research [1, 9].

It is unclear how these anomalies affect the tick’s ability to acquire, maintain, and transmit infectious agents. Domínguez and Bermúdez [4], suggested it does not seem to affect the tick’s ability to find a bloodmeal, as these ticks are often found attached to their hosts. Relatedly, our report of a partially engorged tick demonstrates that the tick can still feed, but further research is needed to understand the reproductive capacity of such malformations. Under laboratory conditions, evidence suggests that external morphological abnormalities can impair some biological abilities in the ticks [12]. Rodríguez-Durán and colleagues observed reduced body weight, oviposition, and fertility in ticks with external malformations, and explained that some malformations affected the feeding efficiency and reproductive capacity of the ticks. While they did not report gynandromorphism, we suspect that this type of malformation could have further impacts on reproductive capacity, oviposition, and fertility in affected ticks.

The relevance of gynandromorphism remains understudied and thus, reporting these anomalies is important for the scientific community. Future research should continue focusing on the morphological diversity of medically and veterinary relevant arthropods in El Salvador, with the goal to better understand the biological and the possible ecological and epidemiological implications it carries.

## Data Availability

No datasets were generated or analysed during the current study.
